# A Highly Sensitive Kinetic Spectrophotometric Method for the Determination of Ascorbic Acid in Pharmaceutical Samples

**Published:** 2014

**Authors:** Masoud Reza Shishehbore, Zahra Aghamiri

**Affiliations:** *Department of Chemistry, Yazd Branch, Islamic Azad University, Yazd, Iran.*

**Keywords:** Kinetic spectrophotometry, Ascorbic acid, Vitamine C, Pharmaceutical samples

## Abstract

In this study, a new reaction system for quantitative determination of ascorbic acid was introduced. The developed method is based on inhibitory effect of ascorbic acid on the Orange G-bromate system. The change in absorbance was followed spectrophotometrically at 478 nm. The dependence of sensitivity on the reaction variables including reagents concentration, temperature and time was investigated. Under optimum experimental conditions, calibration curve was linear over the range 0.7 – 33.5 μg mL^-1^ of ascorbic acid including two linear segments and the relative standard deviations (*n *= 6) for 5.0 and 20.0 μg mL^–1^ of ascorbic acid were 1.08 and 1.02%, respectively. The limit of detection was 0.21 μg mL^−^^1^ of ascorbic acid. The effect of diverse species was also investigated. The developed method was successfully applied for the determination of ascorbic acid in pharmaceutical samples. The results were in a good agreement with those of reference method.

## Introduction

A vitamin is an organic compound that required as a vital nutrient in tiny amounts by an organism. Vitamins are classified as either water-soluble or fat-soluble. Water-soluble vitamins dissolve easily in water and, in general, are readily excreted from the body to the degree that urinary output is a strong predictor of vitamin consumption. Because they are not readily stored, consistent daily intake is important (1). Vitamin C, also known as ascorbic acid (AA), is a water-soluble vitamin. Unlike most mammals and other animals, humans do not have the ability to make their own vitamin C. Therefore, it must obtain through the diet. Since AA has limited stability and may be lost from foods during storage, preparation and cooking, pharmaceutical products of AA can be used as a supplementary source in human diet. The importance of AA related to roles of it in body; AA is required for the synthesis of collagen, it also plays an important role in the synthesis of the neurotransmitter, norepinephrine and carnitine. Moreover, AA is a highly effective antioxidant and may also be able to regenerate other antioxidants such as vitamin E. The daily recommended intake of AA is about 90 mg and upper take is 2000 mg. The deficiency of vitamin C resulted to Scurvy and overdose disease named Vitamin C mega-dosage (2, 3). Moreover, the content of ascorbic acid in biological fluids can be used to evaluate the extent of oxidative stress in human metabolism, this parameter being associated with cancer, diabetes mellitus and other diseases (4).

In view of the great importance and wide use of AA, numerous analytical techniques have been used for the determination of it in different levels and different matrices such as electrochemical (5-8), chromatographic (9-11), atomic absorption spectrometric (12, 13), flow injection (14, 15) and spectrophotometric (16-18) methods. In recent years, electrochemical methods based on electrocatalytic effect of AA on a modified electrode are the most common technique. The methods have disadvantages such as low repeatability, hard operation in preparation of electrode and time consuming. Especially chromatographic methods are expensive and very efficient in AA determination of complex materials such as vegetables and beverage. As spectrophotometric methods are the instrumental methods commonly used in industrial laboratories, a great number of methods have been proposed for the determination of AA (13-16) and other species (17, 18). High sensitivity, sufficient accuracy, simplicity, speed and the necessity of less expensive apparatus make kinetic spectrophotometric method as an attractive method for the determination of trace elements in samples with different matrices such as foods (19, 20), biological and pharmaceutical (21, 22) samples. 

Various reports have been found for the catalytic determination of AA that is based on their oxidation-reduction properties of AA reviewed and summarized in Table 1 (23-37). Some of these methods suffer form limitations such as lack of selectivity, low sensitivity and/or higher limit of detection. Therefore, the need to a low cost, simple, selective and sensitive method for the quantification of AA is obvious. The reference method for the quantification of AA is a titrimetric procedure based on the reduction of 2,6 dichlorophenolindophenol by AA (38).

In this study, a new reaction system for the quantification of AA was proposed. The method is based on inhibitory effect of AA on the reaction of orange-G with bromate. The decolorization of Orange G at 478 nm was used for monitoring the reaction spectrophotometrically. The developed method which has lower detection limit than some catalytic spectrophotometric methods (24-27, 33-35) has been successfully applied for the determination of AA in pharmaceutical samples.

**Table 1:** General characteristic of catalytic spectrophotometric methods for the determination of ascorbic acid

**Reagent**	**Method**	**D.R** **(** **μ** **g mL** ^-1^ **)**	**D.L** **(** **μ** **g mL** ^-1^ **)**	**Real sample**	**Ref.**
Triiodide	FIA ^d^	0.1–40	0.03	Fruits, jam	23
Vanadotungstophosphoric acid-VO_3_^-^	FIA	up to 80	1.0	Vitamine C tablet	24
Toluidine blue	Fixed time	3-35	1.3	Fruits and vegetables	25
NaOH	FIA	1–25, 1–50	0.5, 0.2	Fruit juices and pharmaceuticals	26
Prussian Blue	FIA	0.88- 17.6	0.41	Pharmaceutical	27
Copper (II) phosphate	FIA	0.88–7.04	0.053	Pharmaceutical	28
Fe(III)-1,10-phenanthroline	FIA	0.88–10.6	0.088	Pharmaceutical	29
Ferrozine a	FIA	0.5–10	0.028	Pharmaceutical, juices and urine	30
MnO_4_^-^-H^+^	FIA	up to 200	-	Vitamin C tablet	31
Rhodamine 6G-Cr_2_O_7_^-2^/I	FIA	0.1–4	0.08	Pharmaceutical, tomato, orange	32
Fe(II) - DPPH b	FIA	5.7–600.0	1.7	Pharmaceutical	33
Methyl Orange-HCl/BrO_3_^-^	Fixed time	1.4–211.3	1.3	Pharmaceutical	34
Fe (III) and 2,2′-dipyridyl	FIA	0.5–20	0.22	Rat's tissues	35
Porphyrin-Cu^+2^	FIA	0.1–1000	0.005	Soft drink	36
Fe(II) - (TPTZ) c	FIA	0.014-1.76	0.0042	Pharmaceutical	37
Orange-G‒BrO_3_^-^	Fixed time	0.7–8.3 & 8.3-33.5	0.21	Pharmaceutical, serum and urine	This work

a Ferrozine: 3-(2-pyridyl)-5,6-diphenyl-1,2,4-triazine-4′,4″-disulphonate;

b DPPH: 2,2′-dipyridyl-2-pyridylhydrazone;

c TPTZ: iron(II) with 2,4,6-tripyridyl-s-triazine; ^d^ FIA: Flow injection analysis.

## Experimental

*Apparatus*

A Shimadzu UV-Vis spectrophotometer (160-A, Japan) with two matched 1-cm glass cell was used to measure the absorbance-time changes at fixed wavelength. A thermostated water bath (Heidolph, Germany) was used to keep the temperature of all solutions at the working temperature at 25.0 ± 0.1 °C. A stop-watch was used to record the reaction time.

*Chemicals and reagents*

Doubly distilled water and analytical reagent grade chemicals were used. Ascorbic acid (Merck) stock solution 100.0 μg mL^-1 ^was prepared just before use by dissolving 0.0100 g of AA in water and diluted to the mark in a 100 mL calibrated flask. An appropriate amount of the solution was used for preparing the working solution. A solution of Orange G (6.6 × 10^-4 ^mol L^-1^) was prepared by dissolving 0.2985 g of Orange G (Merck) in water and diluting to 1.0 L with water. Sulfuric acid solution (4.0 mol L^-1^) was prepared by appropriate dilution of conc. sulfuric acid (Merck). A 0.05 mol L^-1^ of potassium bromate solution was prepared by dissolving 8.3504 g of KBrO_3_ (Merck) in water and diluting to 1000 mL in a calibrated flask.

*General procedure*

The inhibited reaction was studied spectrophotometrically by monitoring the change in absorbance of the reaction mixture at 478 nm (λ_max_). For this purpose, to a series of 10 mL volumetric flasks, 0.8 mL of 6.6 × 10^-4 ^mol L^-1^ of Orange-G solution, 2.1 mL of 4.0 mol L^−1^ sulfuric acid solution and the sample or standard solutions containing 7.0 - 335.0 µg of ascorbic acid were added. The solution was mixed and diluted to 8 mL with water. Then, 1.0 mL of 0.05 mol L^−1^ bromate solution was added and diluted to the mark. A time measurement was started just after adding the last drop of the bromate solution. After thorough mixing, a portion of the solution was transferred to a glass cell. The absorbance of inhibited reaction (Δ*A*_s_) was measured against water at λ_max_ and 30 °C for time interval 0.5-3.5 min. The measurement in the absence of ascorbic acid was repeated to obtain the values for the uninhibited reaction (Δ*A*_b_). Finally, the difference in the absorbance change was considered as the response (Δ*A* = Δ*A*_b_ - Δ*A*_s_). The calibration curve was constructed by plotting the response against the ascorbic acid concentration. 

*Procedure of sample preparation*

*Pharmaceutical samples preparation*

Five ascorbic acid tablets (in dose of 250.0 mg) were powdered and mixed thoroughly. An amount corresponding to 250.0 mg of ascorbic acid was weighed, dissolved with 10.0 mL of water and sonicated for 3 min. The sample was filtered through a Whatman filter paper (No. 1), transferred to a 25 mL volumetric flask and diluted to the mark with water. A suitable aliquot of the solution was used for analysis using the procedure. Also, the injection solution (500.0 mg) was diluted in a 1 L volumetric flask. An appropriate amount of the solution was used in each analysis. 

## Results and Discussion

**Scheme 1:**
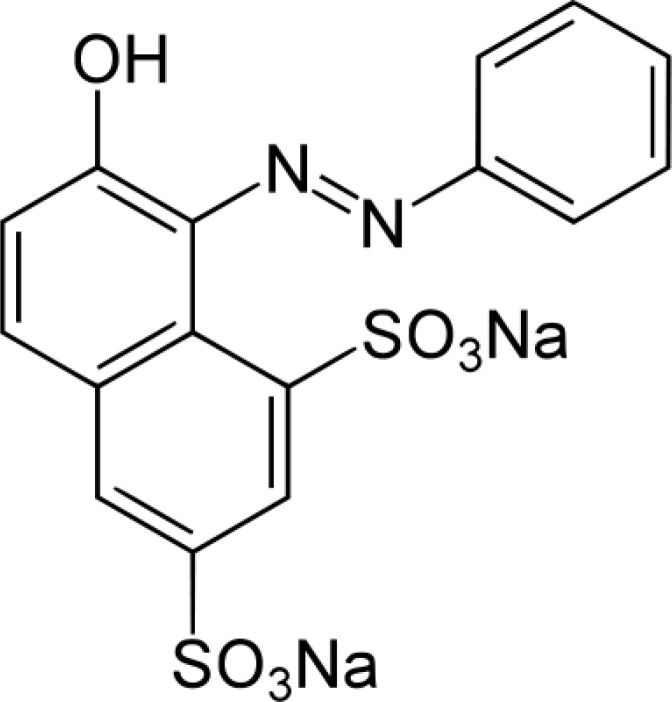
Molecular structure of Orange G.

**Figure 1:**
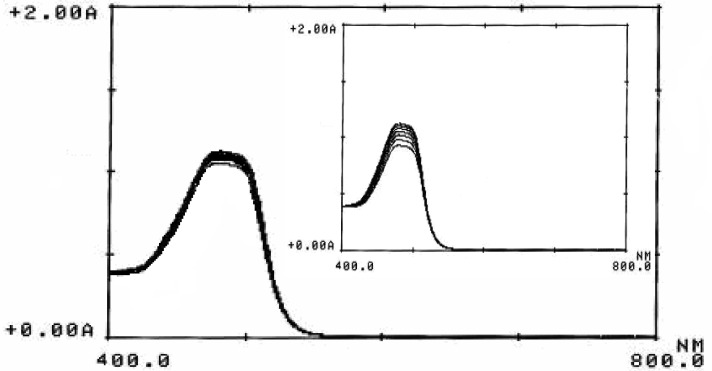
Absorption spectra of the inhibited reaction. (Conditions: Orange G, 6.6 × 10^-5^ mol L^-1^; sulfuric acid, 0.8 mol L^-1^; ascorbic acid, 1.5 µg mL^-1^; bromate, 5.0 × 10^-3^ mol L^-1^; 25 °C and 4.0 min). Inset shows the absorption spectra of the uninhibited reaction

Orange G (see Scheme 1 for molecular structure), a yellowish powder, is a synthetic azo dye. It can be used to stain keratin in histology, color marker to following the electophoresis process, pH indicator, dyeing of textiles, paper and leather and preparing of coloring inks (39, 40). Orange G can be oxidized to a colorless product by oxidizing agents (41). Figure 1 and its inset shown the absorption spectra of the reaction mixture (Orange G, sulfuric acid and bromate) in presence and absence of ascorbic acid, respectively. Comparison of the two spectra indicated that trace amounts of ascorbic acid can be reduce the change in absorbance seriously. Therefore, the proposed reaction system can be used for the determination of ascorbic acid.

The suggested reaction mechanism for Orange G - bromate system may be represented as follow:

The uninhibited reaction that resulted to blank signal (∆*A*_b_) carries out in a cyclic way by these reactions: 

Equation (1) $${\mathrm{Orange G}}_{\left(\mathrm{Red}\right)}+{\mathrm{BrO}}_{3}^{-}+{6H}^{+}\to {\mathrm{Orange G}}_{\left(\mathrm{OX}\right)}+{\mathrm{B1}}^{-}+{3H}_{2}O$$

Equation (2) 2 BrO3-+12H++10Br-→6Br2+6H2O

Equation (3) $${\mathrm{Orange G}}_{\left(\mathrm{Red}\right)}+{\mathrm{Br}}_{2}+H^{+}\to {\mathrm{Orange G}}_{\left(\mathrm{OX}\right)}+{2\mathrm{Br}}^{-}$$

In the presence of AA, reducing agent which reacts slowly with bromate but rapidly with bromine, decolorizing of Orange G reaction was inhibited. AA reacts with bromine according to the following reaction that causes an inhibitory effect on the decolorization of Orange G by bromine:

Equation (4) $${\mathrm{AA}}_{\left(\mathrm{Red}\right)}+{\mathrm{Br}}_{2}+H^{+}\to {2\mathrm{Br}}^{-}+{\mathrm{AA}}_{\left(\mathrm{OX}\right)}$$

Where Red and OX are the reduced and oxidized form of reactant, respectively. AA can be oxidized faster than orange G, AA has an inhibitory effect.

*Optimization of the effective factors*

In orther to obtain the maximum sensitivity as employing the proposed procedure, the effective factors including reagents concentration and reaction conditions must be optimized. The maximum response was considered to obtain the most sensitive results.

*The effect of Orange G concentration*

**Figure 2:**
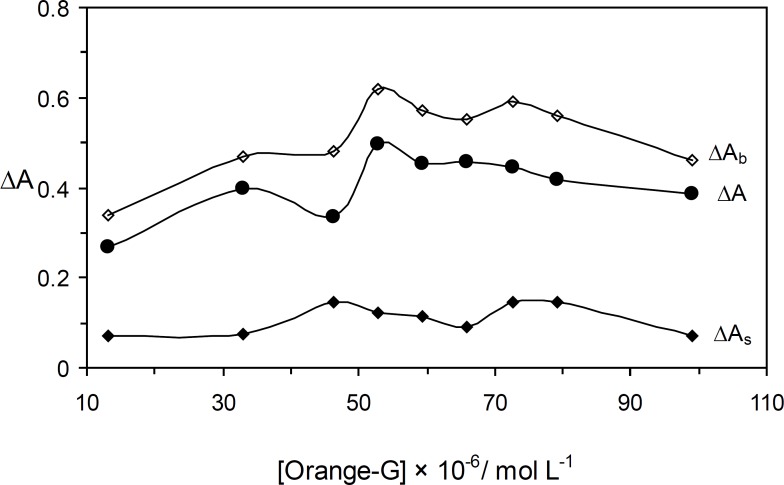
Effect of Orange G concentration on the rate of uninhibited (Δ*A*_b_), inhibited (Δ*A*_s_) reactions and response (Δ*A*). (Conditions: sulfuric acid, 0.8 × 10^-^^3^ mol L^-1^; ascorbic acid, 1.5 μg mL^-1^; bromate, 5.0 × 10^-^^3^ mol L^-1^; 25 °C and 4.0 min).

The effect of Orange G concentration on the rate of reaction was studied over the range 13.2 × 10^-6 ^ – 99.0 × 10^-6 ^ mol L^-1^. As it an be seen in Figure 2, the sensitivity was increased up to 52.8 × 10^-6 ^ mol L^-1^ of Orange G. At higher concentrations, the reaction rate was decreased that may be attributed to the dye aggregation (21). Thus, 52.8 × 10^-6 ^ mol L^-1^ of Orange G as optimum concentration was selected for further study. 

*The effect of sulfuric acid concentration*

**Figure 3:**
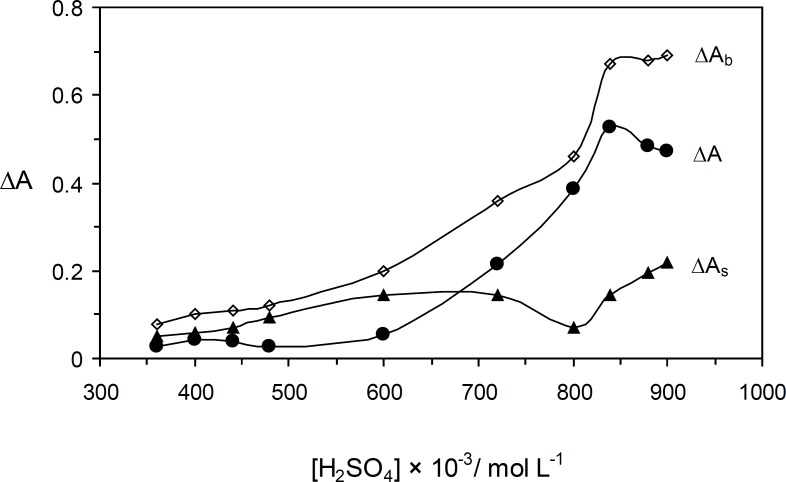
Effect of sulfuric acid concentration on the rate of uninhibited (Δ*A*_b_), inhibited (Δ*A*_s_) reactions and response (Δ*A*). (Conditions: Orange G, 52.8 × 10^-^^6^ mol L^-1^; ascorbic acid, 1.5 µg mL^-1^; bromate, 5.0 × 10^-^^3^ mol L^-1^; 25 °C and 4.0 min).

The effect of the sulfuric acid concentration on the catalyzed and uncatalyzed reactions was studied over the range of 0.36 to 0.90 mol L^-1^ (Figure 3). The maximum sensitivity was obtained at 0.84 mol L^-1^, whereas at higher concentrations the sensitivity was decreased. Protonation of Orange G at higher acid concentrations that make oxidaion quite defficult resulted to the disorder (21). Therefore, 0. 84 mol L^−^^1^ of sulfuric acid was used for further study.

*The effect of bromate concentration*

**Figure 4:**
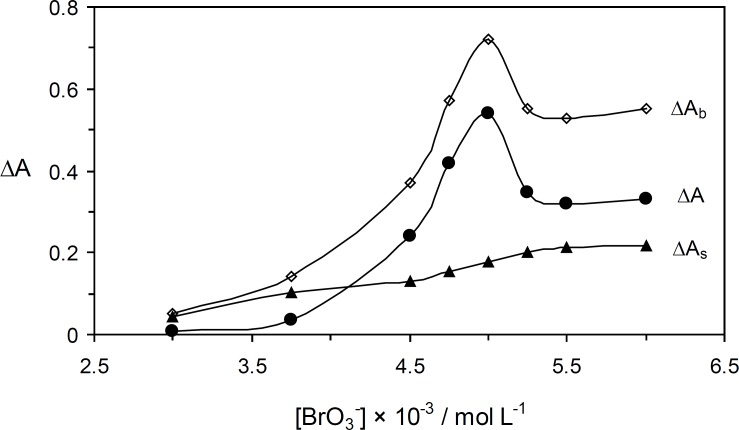
Effect of bromate concentration on the rate of uninhibited (Δ*A*_b_), inhibited (Δ*A*_s_) reactions and response (Δ*A*). (Conditions: Orange G, 52.8 × 10^-^^4^ mol L^-1^; sulfuric acid; 0.84 mol L^-1^; 25 °C and 4.0 min).

The effcet of bromate concentration on the reaction rate was studied in concentration range 3.0× 10^-3 ^ – 6.0 × 10^-3 ^ mol L^-1^. As shown in Figure 4, the net reaction rate was increased up to 5.0 × 10^-3 ^mol L^-1^ which was selected as being the optimum concentration of oxidant.

*The effect of temperature*

**Figure 5:**
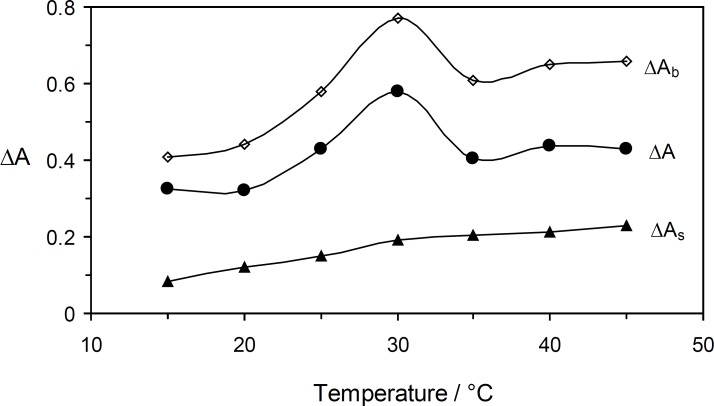
Effect of temperature on the rate of uninhibited (Δ*A*_b_), inhibited (Δ*A*_s_) reactions and response (Δ*A*). (Conditions: Orange G, 52.8 × 10^-^^6^ mol L^-1^; sulfuric acid, 0.84 mol L^-1^; bromate, 5.0 × 10^-^^3^ mol L^-1^ and 4.0 min).

Under optimum experimental conditions, the effect of the temperature on the reaction rate was studied in the range of 15 to 45 °C (Figure 5). Increasing the temperature up to 30 °C caused an increase in the sensitivity, whereas at higher temperatures it decreased. Thus, 30 °C was selected as being the optimum temperature.

*The effect of time*

**Figure 6:**
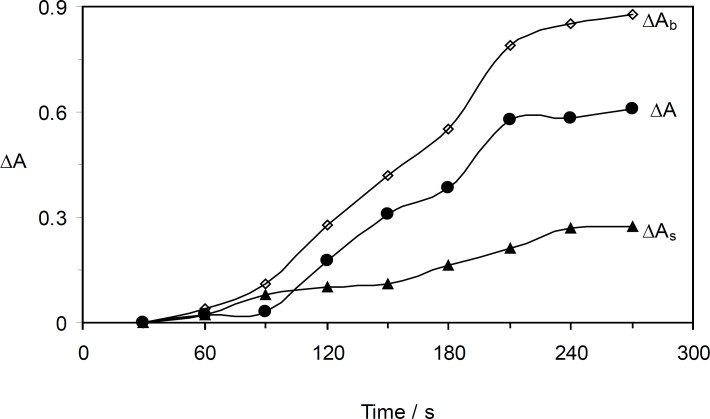
Effect of time on the rate of uninhibited (Δ*A*_b_), inhibited (Δ*A*_s_) reactions and response (Δ*A*). (Conditions: Orange G, 52.8 × 10^-^^6^ mol L^-1^; sulfuric acid, 0.84 mol L^-1^; bromate, 5.0 × 10^-^^3^ mol L^-1^ and 30 °C).

As it can be seen in Figure 6, the optimum time was found by measuring the change in the absorbance during 30 – 270 s. The reaction rate increased up to 210 s, and in longer times the reaction rate was almost constant. Therefore, 210 s was selected for further study.

*Analytical parameters*

*Linearity*

Calibration curve was constructed by plotting the response against AA concentration. Using the recommended procedure and under optimized conditions that outlined above, calibration curve was linear over the range 0.7 – 33.5 μg mL^-1^ of AA including two segments of 0.7 – 8.3 and 8.3 – 33.5 μg mL^-1^. The regression equation of the two segments gaves as equations 5 and 6, respectively.

Equation (5) $$\mathrm{\Delta A}=\mathrm{0.0129}[\mathrm{AA}]+\mathrm{0.5539}(R^{2}=\mathrm{0.9978})$$

Equation (6) $$\mathrm{\Delta A}=\mathrm{0.0043}[\mathrm{AA}]+\mathrm{0.6216}(R^{2}=\mathrm{0.9993})$$

where Δ*A* is the difference in the absorbance between the blank and the sample, [AA] is the ascorbic acid concentration in μg mL^–1^ and R^2^ is the correlation coefficient. 

*Limit of detection (LOD) and precision*

The limit of detection (3*s*_b_/*m*; *s*_b_ is the standard deviation of the blank signal and *m* is the slope of calibration curve) was 0.21 μg mL^−^^1^ of AA for ten replicate determinations. The relative standard deviations (*n* = 6) were 1.08, 1.02% for 5.0 and 20.0 μg mL^−^^1^of AA, respectively.

*Interference investigation*

In order to asses the possible analytical applications of the proposed method, the influence of concomitant species on the determination of ascorbic acid in real samples was studied. The tolerance limit was taken as the concentration which caused an error of 5% in rate of the inhibited reaction with 2.0 μg mL^-1^ of AA. The results were given in Table 2. According to the results, interferences from Na^+^, K^+^, Ca^2+^, Mg^2+^, NH_4_^+^, methaol and ethanol were not found. Cl^–^, Br^–^, I^–^, NO_2_^–^and citric acid have seriously interfering effect that did not found in pharmaceutical samples. The interfering effect of Fe^3+^ was reduced using masking agents. Therefore, the analytical parameters confirms the potential of the proposed method as an alternative for the quantitative determination of AA.

**Table 2:** Tolerance limit for foreign species on the determination of 2.0 μg mL^–1^ of ascorbic acid.

Foreign species	Tolerance limit (W_species_/W_ascorbic acid_)
Na^+^, K^+^, Ca^2+^, Mg^2+^, NH_4_^+^	1000
MeOH, EtOH, Sulfamic acid	1000
Glucose, Saccarose	950
Fructose	900
Zn^2+^, Mn^2+^, Co^2+^	850
Fe^3+ ^a	800
Urea	650
Uric acid, citric acid	320
NO_3_^-^, CN^-^, CO_3_^2-^, SO_4_^2-^, CH_3_CO_2_^-^, F^-^	1000
C_2_O_4_^2-^	900
I^-^, Br^-^, Cl^-^	75
NO_2_^-^	10

a After adding 2 mL of 3% NaF.

*Application in real samples*

Evaluation the reliability and analytical applicability of the developed method makes it potentially useful for the quantitative determination of AA in real samples with different matrices. Pharmaceutical sample preparation was performed using the mentioned procedure. An appropriate amount of the samples were analysed by the recommended procedure and AOAC reference method (38). The results of four replicate determinations were given in Table 3. The obtained results indicated that AA contents by the two procedure are in good agreement together. The precision (RSD%) varies in the range 0.74-0.85% and 0.87-1.26% for AA tablet and injection solution, respectively. The statistical t-test did not show any significant difference between the data obtained from the two methods (for 95% confidence level and four degrees of freedom). Also, the precision of the proposed method and reference method was evaluated using F-test. The precision of the two methods is the same, as obtained results confirm it. Therefore, the developed method can be used for analysis of AA in pharmaceutical samples.

**Table 3:** Determination of ascorbic acid in ascorbic acid tablet and ascorbic acid injection solution using the procedure.

**Sample**	**Proposed method**	**RSD** **(%)**	**AOAC**	**RSD (%)**	**Labled** **(mg/tablet)**	**Statistical test**		**Pharmaceutical Co./ ** **Batch No.**
	**Found** a ** (mg/tablet)**		**Found** a ** (mg/tablet)**			**t-test** b	**F-test** c	
**Ascorbic acid ** **tablet**								
1	248.1 ± 2.1	0.85	246.7 ± 1.9	0.77	250	1.33	1.22	Osvah-Iran/438
2	248.1 ± 2.0	0.80	247.3 ± 1.8	0.73	250	0.80	1.23	
1	243.4 ± 1.8	0.74	242.2 ± 1.9	0.78	250	1.33	1.11	Osvah-Iran/439
2	244.9 ± 1.9	0.77	246.1 ± 2.0	0.81	250	1.26	1.11	
**Ascorbic acid** ** injection solution**								
1	495.5 ± 4.3	0.87	494.5 ± 5.3	1.07	500	0.46	1.52	Daru Pakhsh-Iran/778
2	482.4 ± 4.5	0.93	484.0 ± 4.2	0.87	500	0.71	1.15	
1	493.1 ± 6.2	1.26	495.1 ± 5.6	1.13	500	0.64	1.22	Daru Pakhsh-Iran/781
2	491.0 ± 5.0	1.02	488.3 ± 4.5	0.92	500	1.26	1.23	

a Mean ± standard deviation (n=4)

b Tabulated *t*-value for three degrees of freedom at P(0.95) is 3.18.

c Tabulated *F*-value for three degrees of freedom at P(0.95) is 9.28.

## Conclusion

This study reports a sensitive and relatively selective spectrophotometric method for the detrmination of AA using Orange G as a new reagent. The developed method possesses distinct advantages over other existing methods in cost, simplicity, ease of operation and applicable to real samples. Lower quantitative limit (25-29, 33, 34) and wider linear dynamic range (25, 27-30, 32, 35) are the most important advantages of the proposed method compared to the catalytic spectrophoyometric reported methods in the literature. Moreover, the reliability of the method permits the analysis of pharmaceutical samples with satisfactory results.
